# Opportunistic computed tomography-based sarcopenia screening and mortality after liver transplantation: a systematic review and meta-analysis

**DOI:** 10.1590/1806-9282.20241672

**Published:** 2025-07-07

**Authors:** Daniel Alvarenga Fernandes, Daniel Bohn, Pedro Antune da Silva Pereira, Mila Mucci, Vittor Hugo Andrade Marques, Paula Juliano Lopes de Faria, Elaine Cristina de Ataide, João Rafael Terneira Vicentini, Nelson Marcio Gomes Caserta, Ilka de Fátima Santana Ferreira Boin

**Affiliations:** 1 Universidade Estadual de Campinas, School of Medical Sciences, Department of Anesthesiology, Oncology, and Radiology – Campinas (SP), Brazil.; 2 Universidade Federal de Pelotas, School of Medicine – Pelotas (RS), Brazil.; 3 University of Toronto, Department of Radiology – Toronto (ON), Canada.; 4 Department of Medicine, Santa Casa Hospital – Ribeirão Preto (SP), Brazil.; 5 Faculty of Medicine – Ipatinga (MG), Brazil.; 6 Universidade Estadual de Campinas, School of Medical Sciences, Department of Surgery, Liver Transplant Unit – Campinas (SP), Brazil.; 7 Department of Radiology, Massachusetts General Hospital, Harvard Medical School – Boston (MA), United States.

**Keywords:** Sarcopenia, Psoas muscles, Mortality, Liver transplantation, Multidetector computed tomography

## Abstract

**OBJECTIVE:**

The aim of this study was to investigate the diagnosis of sarcopenia in chronic liver disease by opportunistic computed tomography screening using the Psoas Muscle Index method as a predictor of mortality after liver transplantation.

**METHODS:**

We systematically searched PubMed, Scopus, and Cochrane Central Register of Controlled Trials from July 2014 to July 2024. We restricted inclusion in this meta-analysis to observational studies and clinical trials on adult patients with chronic liver disease diagnosed with sarcopenia by computed tomography undergoing liver transplantation or on a waiting list. We included the one with a larger sample size for studies with overlapping populations. We excluded studies with therapeutic interventions, animal experiments, cell-line studies, editorial pieces, commentaries, review articles, and case reports.

**RESULTS:**

After the removal of duplicate records and ineligible studies, 163 remained and were thoroughly reviewed based on inclusion criteria. Of these, a total of 11 studies were included in qualitative synthesis and four studies were included in quantitative analysis (meta-analysis), comprising 382 patients. Patients with muscle mass loss after liver transplantation diagnosed by computed tomography scan using the Psoas Muscle Index method had a 4.1 times higher risk of death than non-sarcopenic patients (random-effects model: OR 4.1386; 95%CI 2.4215–7.0730; p<0.0001). Interpretatively, a scale with an I2 value close to 0% indicates no heterogeneity. The other criteria also did not reject the hypothesis of homogeneity among the articles.

**CONCLUSIONS:**

Patients with muscle mass loss diagnosed by computed tomography using the Psoas Muscle Index method had a fourfold increased mortality risk after transplantation. The findings reinforce the need to identify sarcopenic patients preoperatively to optimize liver transplantation outcomes. Using the Psoas Muscle Index, an opportunistic diagnosis by computed tomography scan can be helpful in this setting.

## INTRODUCTION

*Sarcopenia* is a known complication occurring in 30–70% of patients with chronic liver disease (CLD)^1,2^ that has emerged as an association with worse outcomes^3,4^. Muscle mass loss is emerging as an independent predictor for the prognosis of patients undergoing liver transplantation^5^. However, the traditional scores used for prognosis in CLD, such as Child-Turcotte-Pug (CTP) and the Model for End-Stage Liver Disease (MELD), do not assess muscle mass loss. There is still no well-established and accepted consensus on a specific computed tomography (CT) scan method with a cutoff point to diagnose sarcopenia^1^. Identification of muscle mass loss in CLD by standardized cutoff point CT diagnostic technique could predict adverse outcomes and help select the best candidates for liver transplantation.

In the case of patients awaiting liver transplantation or transplanted patients, CT scanning would have already been performed due to the underlying disease. Thus, it is possible to diagnose sarcopenia by employing a gold standard examination through an opportunistic diagnosis. Some authors have suggested the need for further studies to incorporate a standardized muscle mass index into a prognostic scale for cirrhotic patients^6,7^.

The Psoas Muscle Index (PMI, cm^2^/m^2^) is calculated using the following formula: psoas muscle area (cm^2^)/the square of the body height (m^2^). Many authors^8–10^ have pointed out the need for methodological standardization to assess sarcopenia in liver transplant candidates and patients with cirrhosis-associated cachexia. In this sense, this systematic review and meta-analysis aimed to investigate the diagnosis of sarcopenia in CLD by opportunistic CT using the PMI method as a predictor of mortality after liver transplantation.

## METHODS

### Protocol and registration

We performed this systematic review and meta-analysis in accordance with the Cochrane Collaboration and the Preferred Reporting Items for Systematic Reviews and Meta-Analysis (PRISMA) statement guidelines^11^. Before beginning the process, we registered the protocol with the International Prospective Register of Systematic Reviews (PROSPERO) per PRISMA guidelines.

### Eligibility criteria

We restricted inclusion in this meta-analysis to studies that met all the following eligibility criteria: (1) observational studies and clinical trials;(2) patients with CLD diagnosed with sarcopenia by CT; (3) patients with CLD diagnosed with sarcopenia by CT undergoing liver transplantation or on a waiting list; and (4) adults. In addition, we included studies that reported any of the clinical outcomes of interest. We included the one with a larger sample size for studies with overlapping populations. We excluded studies conducted on (1) patients under 18 years of age; (2) studies including a diagnosis of sarcopenia only by other methods besides CT; (3) studies with therapeutic intervention; (4) animal experiments, cell-line studies, and editorial pieces; and (5) commentaries, review articles, and case reports.

### Search strategy and data extraction

We obtained the references included in this review from three independent groups (Group 1: DAF, DB; Group 2: MM, VHAM; Group 3: PASP, PJLF) by searching the databases PubMed, Scopus, and Cochrane Central Register of Controlled Trials from July 2014 to July 2024. We used the following Medical Subject Headings (MeSH) terms for the search: (Sarcopenia OR "muscle mass" OR "psoas muscle") AND ("chronic liver disease" OR CLD OR cirrhosis OR "liver transplantation" OR "liver transplant") AND ("Computed Tomography" OR CT OR "psoas muscle"). The references from all included studies, previous systematic reviews, and meta-analyses were also searched manually for any additional studies.

### Quality assessment

We investigated publication bias by funnel-plot analysis of point estimates according to study weights. A natural way to visualize the evidence of possible publication bias effects is the funnel plot, which plots each study treatment effect (x-axis) against a measure of its variability (y-axis); usually, this is the standard error. The funnel plot shows an idea of whether there is any treatment effect dependent on precision.

### Statistical analysis

Cochran's Q test and I^2^ statistics assessed heterogeneity; p-values inferior to 0.10 and I^2^>25% were considered significant. We used a fixed-effect model for outcomes with low heterogeneity (I^2^<25%). We calculated standard effect estimates and random effects (odds ratio [OR]) for meta-analysis with binary outcome data. Clustering was performed by the Mantel-Haenszel method. We used R Core Team (Vienna, Austria) software for statistical analysis^12,13^.

## RESULTS

### Study selection and characteristics

The initial search yielded 1,414 results. After removing duplicates (n=497) and excluding records based on title and abstract (n=754), we assessed 163 studies in full text for eligibility. Of these, we excluded studies for the following reasons: use of diagnostic methods other than CT (n=52); use of CT but not the PMI (n=91); outcomes not of interest (n=4); participants under 18 years of age (n=3); and therapeutic interventions (n=2). Ultimately, we included 11 studies in the qualitative synthesis and four in the quantitative meta-analysis, comprising 382 patients. Definitions of sarcopenia by CT using PMI varied between studies, and we presented them in Table 1 ^14–22,5,10^.

**Table 1:** Included studies analyzing sarcopenia in chronic liver disease by opportunistic computed tomography screening using the Psoas Muscle Index method as a predictor of mortality after liver transplantation.

First author	Year	Country	Patients	Cutoff
Malamutmann et al.^14^	2021	Germany	265	Not reported
Alconchel et al.^15^	2020	Spain	57	Not reported
Hentschel et al.^16^	2022	Germany	65	Male: ≤5.0 cm^2^/m^2^ Female: <4.2 cm^2^/m^2^
Esser et al.^5^	2019	Austria	186	Male: <6.36 cm^2^/m^2^ Female: <3.92 cm^2^/m^2^
Hou et al.^17^	2020	China	251	Male: 3.5 cm^2^/m^2^ Female: 2.6 cm^2^/m^2^
Kalafateli et al.^18^	2017	UK	232	Male: 340 mm^2^/m^2^ Female: 264 mm^2^/m^2^
Ebadi et al.^10^	2018	USA	353	Male: <5.1 cm^2^/m^2^ Female: <4.3 cm^2^/m^2^
Wu et al.^19^	2021	China	271	Male: Not assessable Female: 2.63 cm^2^/m^2^
Izumi et al.^20^	2016	Japan	47	Male: 612.5 mm^2^/m^2^ Female: 442.9 mm^2^/m^2^
Tan et al.^21^	2022	China	82	Male: 6.25 cm^2^/m^2^ Female: Not assessable
Hamaguchi et al.^22^	2014	Japan	200	Male: 6.868 cm^2^/m^2^ Female: 4.117 cm^2^/m^2^

### Pooled analysis of all studies

**Figure 1:**
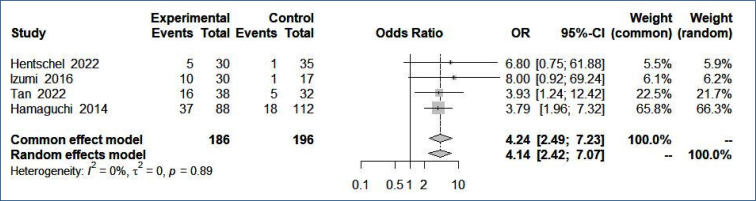
Forest plot to study the effect of sarcopenia on death after liver transplantation by computed tomography scan using the Psoas Muscle Index.

For the summary estimate of the effect measure (in this case, OR), we used the fixed-effects model and the Mantel-Haenszel method. This method was based on the selected studies presenting small sample sizes. Patients with muscle mass loss after liver transplantation diagnosed by CT scan using the PMI method had a 4.1 times higher risk of death than non-sarcopenic patients (random-effects model: OR 4.1386; 95%CI 2.4215–7.0730; p<0.0001). The forest plot studying the effect of sarcopenia on death after liver transplantation by CT scan using the PMI is depicted in Figure 1.

### Quality assessment

Heterogeneity among studies was assessed by visually examining the graphs, the I^2^ statistic, Cochran's Q test, and Tau^2^. Interpretatively, a scale with an I^2^ value close to 0% indicates no heterogeneity. The other criteria also did not reject the hypothesis of homogeneity among the articles.

**Figure 2:**
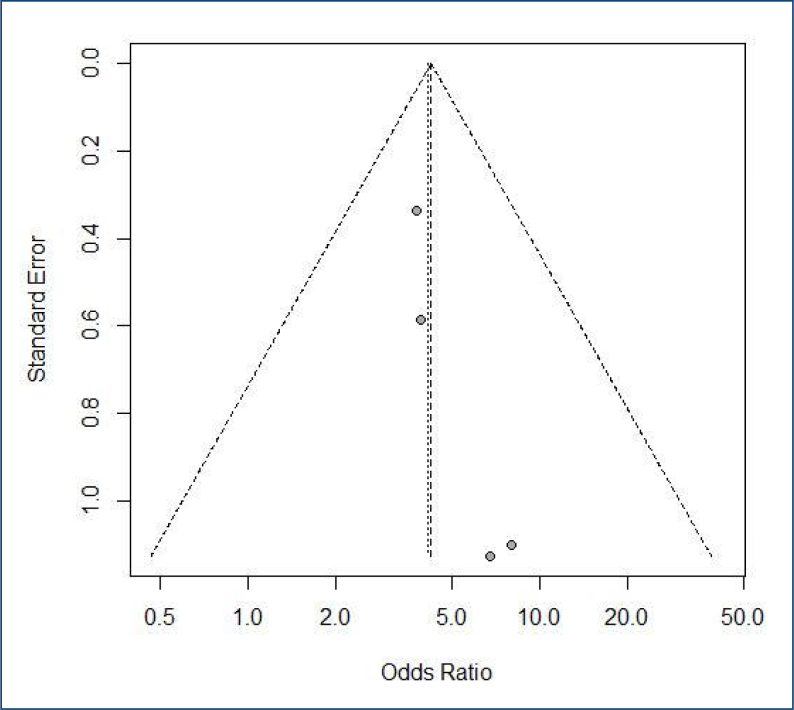
Funnel plot to study the effect of sarcopenia on death after liver transplantation by computed tomography scan using the Psoas Muscle Index

A funnel plot can identify publication bias. The widest part of the funnel, where the least accurate studies are, show two studies, possibly because they are the smallest (Figure 2).

## DISCUSSION

This systematic review and meta-analysis investigated the opportunistic CT diagnosis of sarcopenia using the PMI methodology in patients with CLD undergoing liver transplantation and the association with postoperative mortality. According to our results, patients with muscle mass loss after liver transplantation diagnosed by CT scan using the PMI method had a 4.1-fold higher risk of death than non-sarcopenic patients. The analysis of muscle mass loss exclusively by the PMI method has not yet been performed in previous systematic reviews with meta-analysis. However, other meta-analyses with other associated diagnostic methods have highlighted the harmful effects of sarcopenia on health outcomes^6,7,23,8^.

Chang et al. published a recent meta-analytic review paper addressing mortality in cirrhotic patients and loss of muscle mass. Increased mortality was found before liver transplantation—already-transplanted patients were excluded from the study^23^. Therefore, post-transplant mortality was not assessed in that study. A recent meta-analysis and systematic review evaluating post-transplant mortality and opportunistic CT-based sarcopenia has not been performed, which our study investigated by compiling the most updated articles. Additionally, we based our results on findings using CT with the same method for assessing sarcopenia, the PMI, in cross sections at a specific anatomical level (third lumbar vertebra, L3), which allowed a meta-analysis with low statistical heterogeneity—i^2^=0.0%. Our results corroborate the negative outcome of higher mortality associated with loss of muscle mass following liver transplantation. These findings support future standardization in the imaging evaluation of sarcopenia.

The literature does not define consensus cutoff points for defining sarcopenia based on cross-sectional imaging^8–10^. Most articles define sarcopenia using the muscle area or skeletal muscle index of the lowest quartile in the patient group—below the 5th percentile or less than two standard deviation mean in healthy donor/adult groups^19^. Kalafateli et al. highlighted that there are no defined cut-point values of sarcopenia by PMI for cirrhotic patients^18^. In this sense, as shown in Table 1 (PMI values in patients with CLD and cutoff points in different studies), our findings provide a compilation of data. Our data can be compared with cutoff points in other populations and can be used in future studies to develop a more standardized method for an opportunistic diagnosis of sarcopenia on CT. Additional work is needed to develop valid and clinically relevant measures of sarcopenia and to make associations with other consolidated and new parameters in patients with CLD awaiting or undergoing liver transplantation.

The assessment of muscle mass could be used to better select patients for liver transplantation. The growth and practical implementation of opportunistic CT screening involves discussions on the knowledge and acceptance of this methodology among different clinical medical specialties, surgeons, and radiologists. The cost–benefit ratio and reimbursement for imaging professionals can be essential aspects to be worked on in this gradual growth process in the medical field, including, for example, the review of diagnostic opportunities generated by artificial intelligence^24,25^. The value and potential of these parameters in preoperative clinical and surgical stratification and as a prognostic marker have increasingly revealed their attractiveness as economic results for payers and comprehensive and multidisciplinary healthcare systems focused on excellence. They are personalized to the patient and used in strategies for specific population groups, such as the liver transplant group at our institution.

The limitations of our study include the fact that from the final 11 studies in our systematic qualitative review, only four could be pooled and included in the quantitative analysis (meta-analysis). However, these findings provide a solid platform for future research in this area. Thus, it was not possible to assess the outcome of infection in sarcopenic patients with CLD nor the correlation between infections in these patients and mortality. Chang et al. suggested that mortality might be more related to sepsis than liver failure in cirrhotic patients^23^. The association of muscle mass loss with the length of hospital stay by CT scan using the PMI method also cannot be assessed due to the lack of original studies with this information.

## CONCLUSION

Our meta-analysis highlights that patients with muscle mass loss diagnosed by CT using the PMI method had a fourfold increased risk of mortality after transplantation. The findings reinforce the need to identify sarcopenic patients preoperatively to optimize liver transplant outcomes. Using the PMI, an opportunistic diagnosis by CT can be helpful in this setting.

## Data Availability

The datasets generated and/or analyzed during the current study are available from the corresponding author upon reasonable request.
